# Supplementary material for: Attenuation of SCI-Induced Hypersensitivity by Intensive Locomotor Training and Recombinant GABAergic Cells
Source: Bioengineering (Basel). 2023 Jan 9;10(1):84. doi: 10.3390/bioengineering10010084 (PMC9854592; doi:10.3390/bioengineering10010084)
Supplement: Supplementary file 1 [file bioengineering-10-00084-s001.zip › bioengineering-2104935-supplementary.pdf]

Supplementary Materials

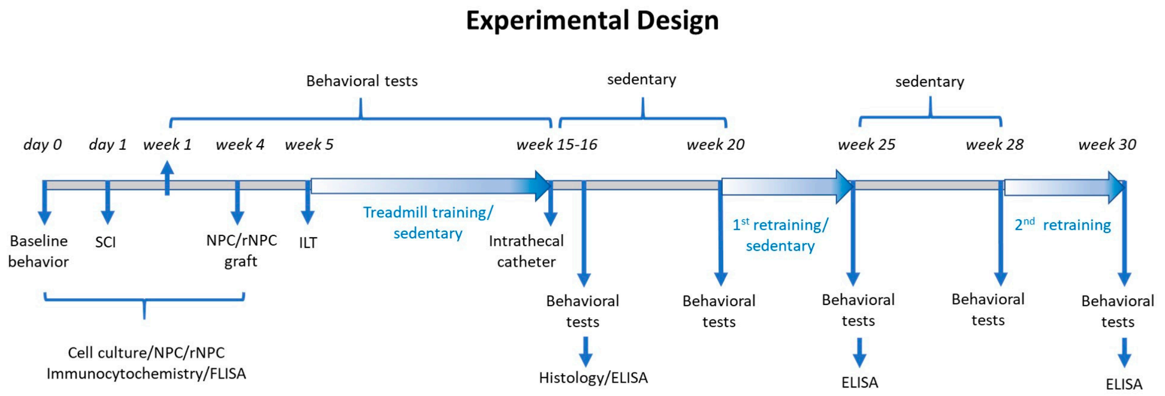

Figure S1. Experimental design and timeline of the study.

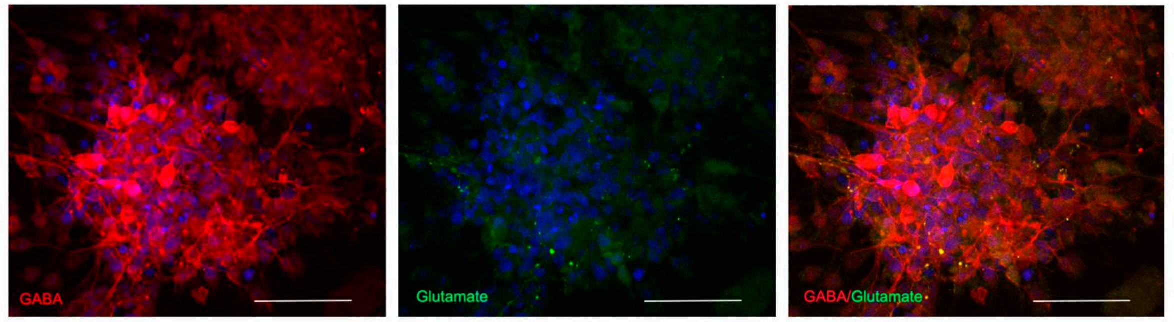

Figure S2. Immunocytochemical analysis of NPCs culture for the presence of GABA (red) and glutamate (green) markers. DAPI used for nuclear staining.

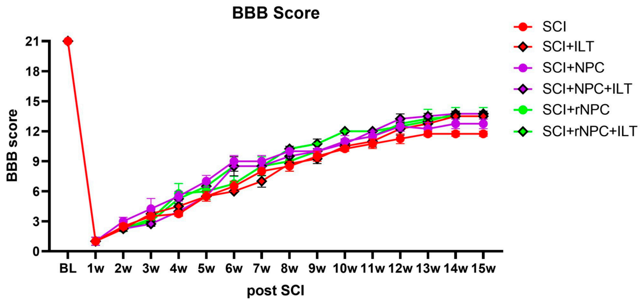

Figure S3. Locomotor scores of animals after SCI with different treatments. N = 10 (SCI, NPC, rNPC), 16(ILT, ILT+NPC, ILT+rNPC).

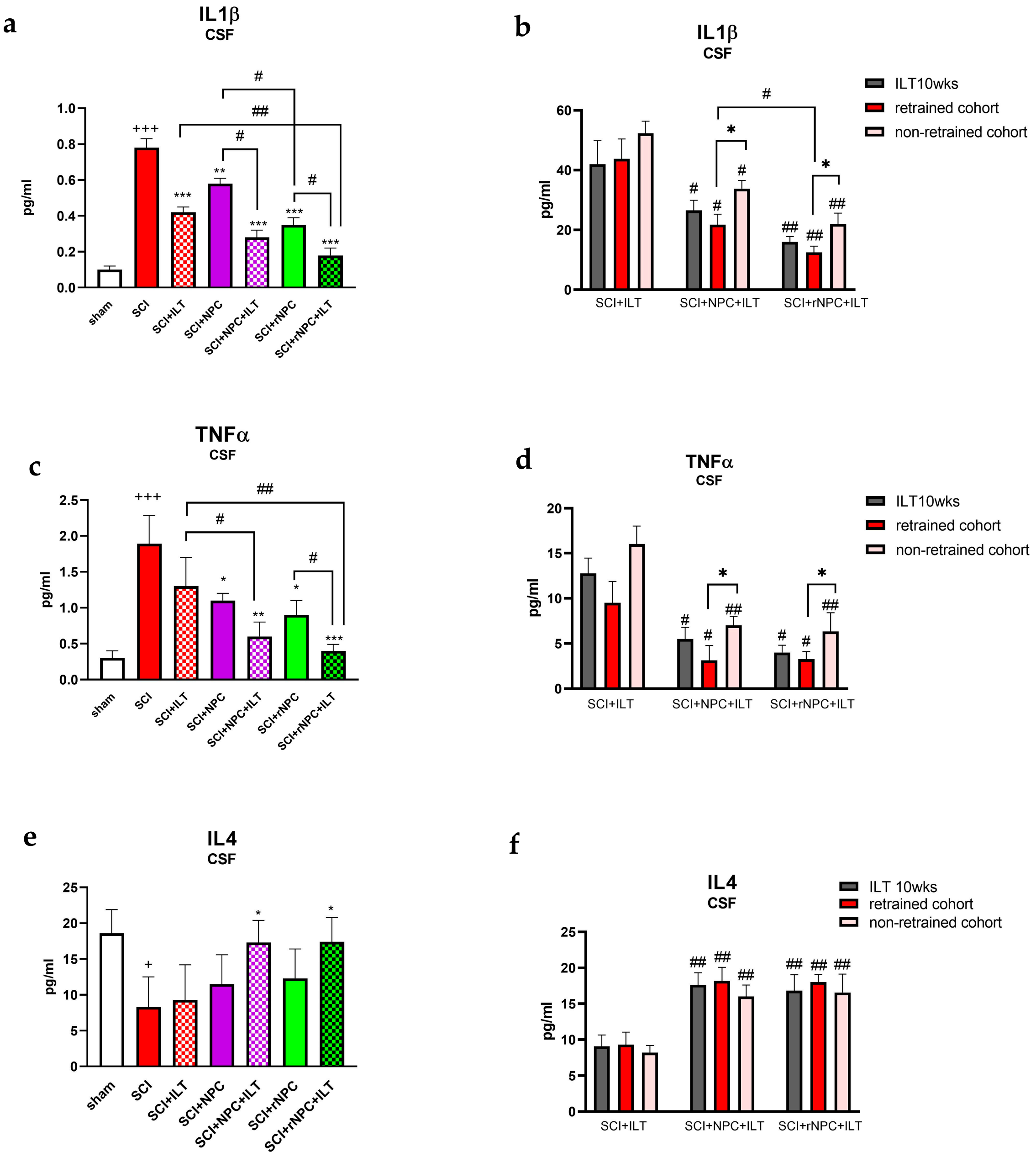

Figure S4. ELISA analysis of IL1β (a, b) TNF*α* (c, d), and IL4 (e, f) in the CSF of animals with different treatments after 10 weeks of ILT/15 weeks post-SCI (left column) and in retrained and non-retrained cohorts (right column). (a, c, e) +$p < 0.05$, +++$p < 0.001$ vs. sham, *$p < 0.05$, **$p < 0.01$, ***$p < 0.001$ vs. SCI, #$p < 0.05$, ##$p < 0.01$, between indicated groups. (b, d, f) *$p < 0.05$ during training/retraining/sedentary periods within each treatment group. #$p < 0.05$ for SCI+NPC+ILT and SCI+rNPC+ILT vs. respective training periods in SCI+ILT group and between indicated groups. n = 3–4/group.
